# Supplementary material for: A simple micropump based on a freeze-dried superabsorbent polymer for multiplex solution processing in disposable devices
Source: R Soc Open Sci. 2019 Mar 27;6(3):182213. doi: 10.1098/rsos.182213 (PMC6458371; doi:10.1098/rsos.182213)
Supplement: Supplementary material [file rsos182213supp1.docx]

**Electronic supplementary material**

**A simple micropump based on a freeze-dried superabsorbent polymer for multiplex solution processing in disposable devices**

Gokul Chandra Biswas^1‡^*, Md. Mohosin Rana^1^, Takekoshi Kazuhiro^2^ and Hiroaki Suzuki^1^

*^1^Graduate School of Pure and Applied Science, University of Tsukuba, Tsukuba, Japan.*

*^2^Faculty of Medicine, University of Tsukuba, 1-1-1 Tennodai, 305-8575 Tsukuba, Japan*

**Email: gcbiswas-geb@sust.edu*

*‡ Present address: School of Life Sciences, Shahjalal University of Science and Technology, Sylhet, Bangladesh.*

**Measurement of *A_c_*, *A_r_* and flow rate**

*A_c_* of the SAP disc was measured using a slightly modified tea-bag method [1,2]. We made small (2 cm × 1 cm) tea bags into which polymer discs (5 mg weight) were inserted. The bags were then completely immersed in the liquid under examination. After 1 hour, the bags were suspended in air to remove any traces of non-absorbed solution until no solution was dropping. In the case of the viscous fluids (APTES, saliva and BSA), we modified the method slightly. We used a small plastic cup containing a sample solution (500 µL) and placed a small piece of a polymer disc into the solution. The polymer disc was allowed to absorb the solution for 1 hour. The solution with the disc was then deposited on a tissue paper for a further hour, to remove the non-absorbed solution. To measure the *A_c_* values of filter paper, cotton thread, tissue paper, cotton, and synthetic sponge, we immersed them completely in distilled water in separate plastic cups and kept for 10 min (observing the time required to gain the maximum absorbency). The wet materials were then put on tissue paper for 2 min to remove excess water. The time of removing the non-absorbed solution was kept shorter to prevent the evaporation and leaching loss of the absorbents.

*A_r_* was determined by measuring the time for the disc to absorb a solution of 8 µL through the flow channel of the device shown in Fig. 2 and S2. The ratio of the solution volume with respect to the dry weight of the disc was much smaller than *A_c_* for the solution to be absorbed. *A_r_* values of the other materials were measured in the same manner.

Pumping performance (figure 3A inset) was characterized by observing the displacement of the water meniscus in a silicone tube with an inner diameter of 500 µm connected to the inlet of the flow channel of the device shown in figure 2. The connection joint was sealed with 1-component RTV KE4 (Shin-Etsu Chemical, Tokyo, Japan), to prevent any leaking. Water (~50 μL) was first loaded in the entire space including the pumping chamber, flow channel of the device, and the tube connected to the device. The polymer disc (~5 mg) was then placed in the pumping chamber, and the movement of the meniscus was observed. The displacement of the meniscus was analyzed using the ImageJ software. The device performance was also assessed by changing the SP disc and the dimensions of the flow channel. In one case (figure S6A), the disc weight was ~35 mg and both the width and the height were 500 µm. In the other case, to slow the pumping (figure S6B), the disc weight was set to ~5 mg and the width and height were 200 µm and 50 µm, respectively. The length of the flow channel was fixed in all cases (4.5 mm). All the experiments were conducted at room temperature.

**Structure and fabrication of the device for glucose detection**

For the detection of glucose, we fabricated a device shown in Fig. S8†. In addition to the micropumps and valves shown in figure 5, a three-electrode system was formed. The working and auxiliary electrodes were formed with platinum, and the reference electrode was Ag/AgCl. To form the Ag/AgCl electrode, a silver layer was formed on a designated area of the platinum pattern (300-nm thick platinum thin layer with an underneath adhesive layer of 50-nm thick chromium), along with the working and auxiliary electrodes. The metals were deposited by sputtering and were patterned by lift-off. An insulating layer was formed on the silver pattern with a positive photoresist, and six pinholes (40 µm in diameter) were formed there to make the active areas of the Ag/AgCl electrode. The exposed silver areas were then immersed in a gently stirred 0.1 M KCl solution under a constant current (50 nA) for 20 min to deposit AgCl into the pinhole areas. The potential of the Ag/AgCl electrode was checked using a commercial Ag/AgCl electrode as a standard. Glucose oxidase (GOD) was immobilized on the platinum working electrode. To this end, GOD was first mixed with 5 µl of 0.5% (w/v) BSA solution, 5 µl of 0.5% (v/v) glutaraldehyde solution, and 10 µl of PBS. The mixture was immediately placed on the working electrode, and the reaction was allowed to proceed for 10 min. Finally, the membrane with immobilized GOD was immersed in a 0.1 M glycine solution for 1 hour. One of the reaction products, hydrogen peroxide, was detected electrochemically on the working electrodes under a polarized condition (+0.7 V vs. on-chip Ag/AgCl).


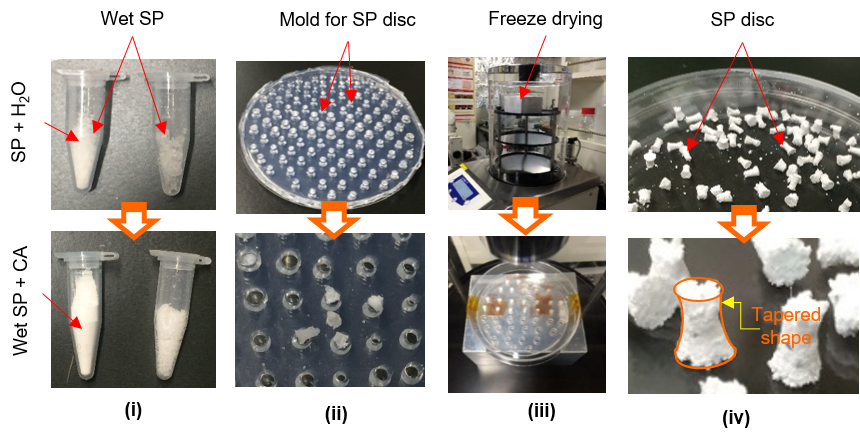


**Figure S1. Formation of the SAP discs.** (i) Wetting and mixing of SP and CA particles. (ii) Injection of the composite into the PDMS mold. (iii) Freeze-drying of the composite. (iv) Freeze-dried SP discs removed from the mold.


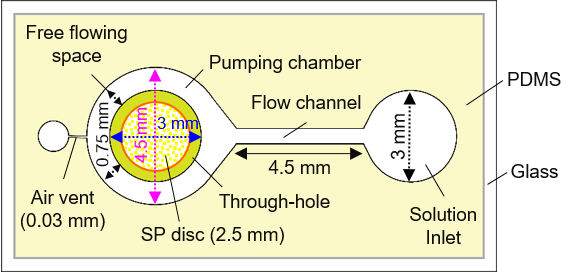


**Figure S2.** Dimensions of the structures in the device shown in figure 2B.

**
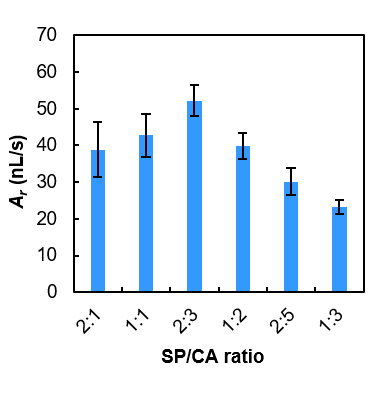
**

**Figure S3.** Dependence of *A_r_* of water on the SP/CA ratio. Error bars indicate standard deviations (number of replication, n=3).

**
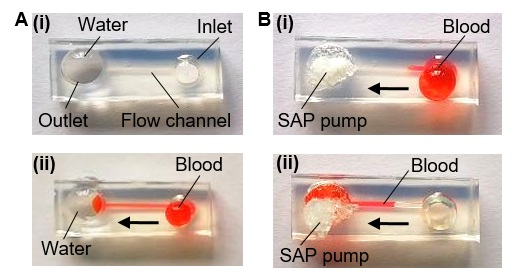
**

**Figure S4.** Transport of blood using the intermediate working solution and the SAP pump. (A) Movement of blood in water by capillary action. (i) Flow channel filled with water, (ii) Blood flowed in the water from the inlet towards the outlet. (B) Blood transport by the passive driven force of SAP pump. (i) Placement of a blood drop on water at the inlet and starting of the blood transport with the water-flow by the SAP, (ii) Absorption of total blood and prevention of reverse-flow by SAP’s gelation. Black arrow indicates the direction of blood flow.

**
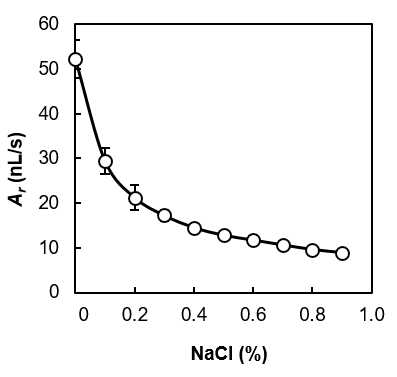
**

**Figure S5.** Dependence of *A_r_* of the SP disc on the NaCl concentration.

**
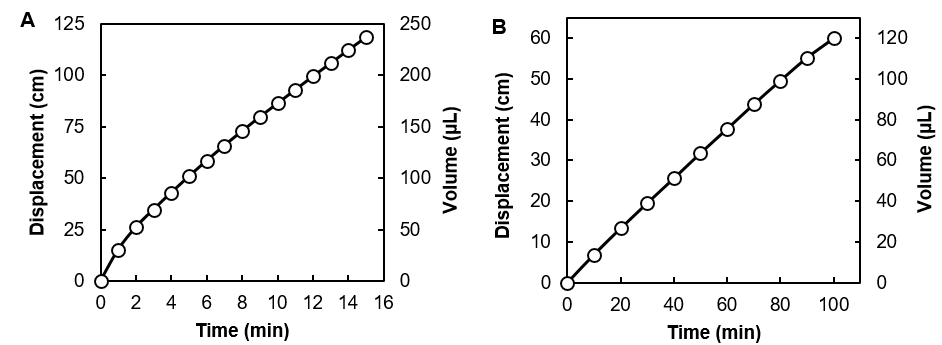
**

**Figure S6.** Time evolution of the displacement of the water meniscus or volume of water absorbed by the SP disc. (A) Disc weight: 35 mg. SP/CA = 2/3. The width and height of the flow channel were 500 µm and 500 µm, respectively. (B) Disc weight: 5 mg. SP/CA = 1/3. The width and height of the flow channel were 200 µm and 50 µm, respectively.


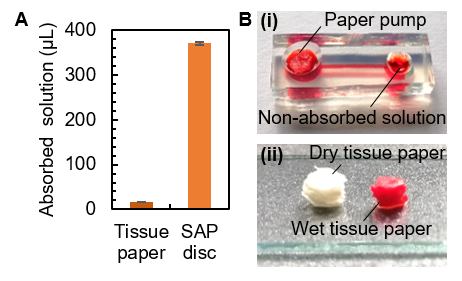


**Figure S7.** Performance of the paper pump. (A) Comparative solution transport performance of paper and SAP pump. Error bars indicate standard deviations (number of replication, n=3). (B) Reverse-flow phenomena of paper pump. (i) Paper left some solution non-absorbed when it reached to the maximum absorption point. (ii) Paper material before (dry) and after (wet) the pumping.

**
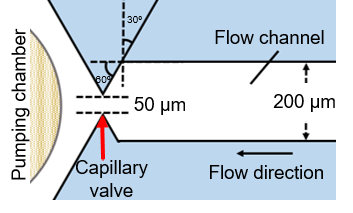
**

**Figure S8.** Structure of the capillary valve used in the device shown in figure 4.

**
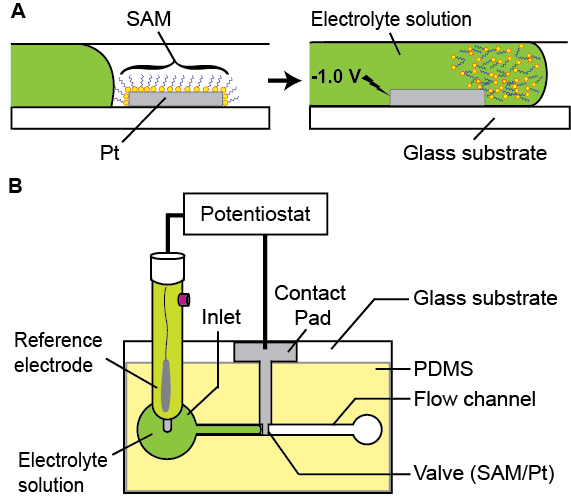
**

**Figure S9.** Switchable hydrophobic valve. (A) Structure and operation of the valve. Without applying a potential, an aqueous solution stops at the hydrophobic valve. By applying an appropriate potential to the platinum electrode, the SAM is removed reductively and the solution passes the electrode area and moves forward in the flow channel. (B) Setup used for the operation of the valve.


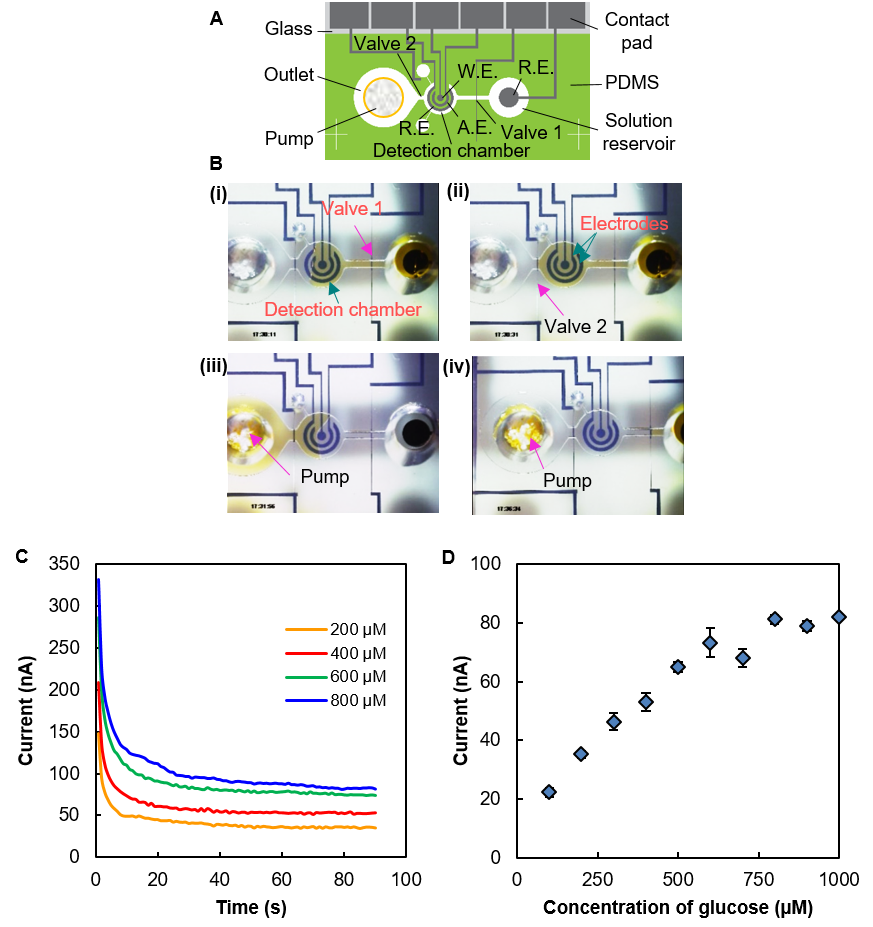


**Figure S10.** Electrochemical detection of glucose. (A) Top view of the device. (B) Transport of an analyte solution. (i) The solution was injected into the detection chamber by opening Valve 1. (ii) The detection chamber was filled with the solution. (iii), (iv) The solution was absorbed into the SAP disc after opening Valve 2. W.E., A.E. and R.E. indicate the working, auxiliary, and reference electrodes, respectively. **(**C) Time courses of current recorded with glucose solutions of different concentrations. (D) Dependence of the current on glucose concentration.


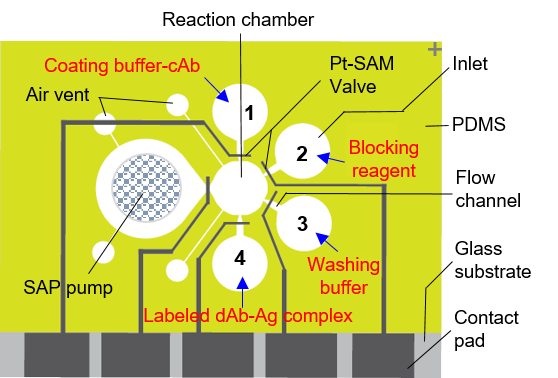


**Figure S11**. Layout of a prospective bioassay device for automatic processing of multiple solutions. The valve at inlet could control the sequential injection of the component solutions such as coating buffer, capture antibody (cAb), blocking reagent, washing buffer and conjugated detection antibody (dAb)-antigen (Ag) complex in the reaction chamber. After the incubation in the reaction zone, the processed solutions could be removed with the SAP pump by opening the valve before the pumping chamber. The numbers inside the photo represents the chronology of steps in assay procedure (e.g coating, blocking, washing and signal generation).

**References**

1. Zohuriaan-Mehr MJ, Kabiri K. 2008 Superabsorbent Polymer Materials: A Review. *Iran. Polym. J.* **17**, 451–477. (doi:http://journal.ippi.ac.ir/search.php)

2. Buchholz FL, Peppas NA. 1994 *Superabsorbent Polymers Science and Technology*. Washington, DC: American Chemical Society. (doi:10.1021/bk-1994-0573)
